# Role of Human Milk Microbiota in Infant Neurodevelopment: Mechanisms and Clinical Implications

**DOI:** 10.3390/children11121476

**Published:** 2024-11-30

**Authors:** Valentina Biagioli, Vincenzo Sortino, Raffaele Falsaperla, Pasquale Striano

**Affiliations:** 1 Department of Neurosciences, Rehabilitation, Ophthalmology, Genetics, Maternal and Child Health, University of Genoa, 16126 Genoa, Italy; pstriano@unige.it; 2 Unit of Pediatrics and Pediatric Emergency, Azienda Ospedaliero-Universitaria Policlinico “Rodolico-San Marco”, San Marco Hospital, University of Catania, 95123 Catania, Italy; v.sortino@policlinico.unict.it; 3 Department of Medical Science-Pediatrics, University of Ferrara, 44124 Ferrara, Italy; raffaele.falsaperla@unife.it; 4 Pediatric Neurology and Neuromuscular Diseases Unit, IRCCS Giannina Gaslini Full Member of EPICARE, 16121-16167 Genoa, Italy

**Keywords:** breastfeeding, human milk oligosaccharides, microbiota–gut–brain axis, preterm infants, malnutrition

## Abstract

Background: Human milk (HM) is recognized as an ideal source of nutrition for newborns; as a result, its multiple bioactive molecules can support the growth of healthy newborns and reduce the risk of mortality and diseases such as asthma, respiratory infections, diabetes (type 1 and 2), and gastrointestinal disorders such as ulcerative colitis and Crohn’s disease. Furthermore, it can reduce the severity of necrotizing enterocolitis (NEC) in preterm infants. Moreover, human milk oligosaccharides (HMOs) present in breast milk show an immunomodulatory, prebiotic, and neurodevelopmental effect that supports the microbiota–gut–brain axis. Material and methods: This study examined the state-of-the-art research, using keywords such as “breastfeeding”, “human milk oligosaccharides”, “microbiota–gut–brain axis”, “infants”, and “malnutrition”. The literature review was conducted by selecting articles between 2013 and 2024, as the most recent ones. The databases used were Web Science, PubMed, and Scopus. Results: We found multiple studies examining the composition of HM and infant formula (IF). However, further longitudinal studies and randomized control trials (RCTs) are needed to better understand the clinical outcomes that bioactive components exert on healthy and hospitalized children and how, in conditions of malnutrition, it is necessary to support the growth of the newborn. Conclusions: In this review, we affirm the importance of human milk and, through it, the modulation of the microbiota and the neuroprotective role in newborns, determining the health of the following years of life.

## 1. Introduction

The first days of life are essential in the development and concretization of the social, cognitive, and emotional domains of the newborn. In these great stages of acquisition, the development of another “invisible organ” occurs, and this is our microbiota [1]. The newborn’s microbiota in the first thousand days of life will undergo massive changes that will be decisive for many years. In this delicate window of opportunity, breastfeeding not only supports the growth of a newborn but also creates a massive passage of microbes from the maternal intestine, thanks to the entero-mammary circulation, which then reaches the intestine of the newborn. The advent of “omic” sciences and new technologies have allowed us to understand more deeply the importance of the microbiota and its regulatory factors, such rural or industrial environment, the pregnant woman’s diet, the type of birth, breastfeeding, weaning, early skin-to-skin contact, or even the use of antibiotics [2]. To ensure the growth, neurodevelopment, and best long-term health of a newborn, HM and its nutrition are the key [3]. Recent data demonstrated that HMOs (the main fraction of HM) not only have a prebiotic, immunomodulatory function and enhance brain development by the microbiota–gut–brain axis [4] but studies on murine models with necrotizing enterocolitis (NEC) have shown how HMOs prevent morbidity and mortality [5]. Furthermore, Vazquez et al. [6] demonstrated that, in animal models, the intake of HMOs, particularly 2′-fucosyllactose (2′-FL), improved learning and memory [7]. However, the intake of prebiotics and, in particular, probiotics in preterm infants is still a highly debated issue. In preterm infants in NICU settings with intestinal immaturity and a mucosal barrier still in development, the intake of probiotics could lead, following intestinal permeability, to a translocation of the same with a risk of sepsis [8,9,10]. For these reasons, this review aims to promote the importance of future studies that can clarify the potential benefit of such supplements in preterm infants. Furthermore, it aims to underline not only the importance of breast milk as a gold standard for child health but as a key modulator for neurodevelopment via the microbiota–gut–brain axis (MGBA).

## 2. Materials and Methods

**Figure 1:**
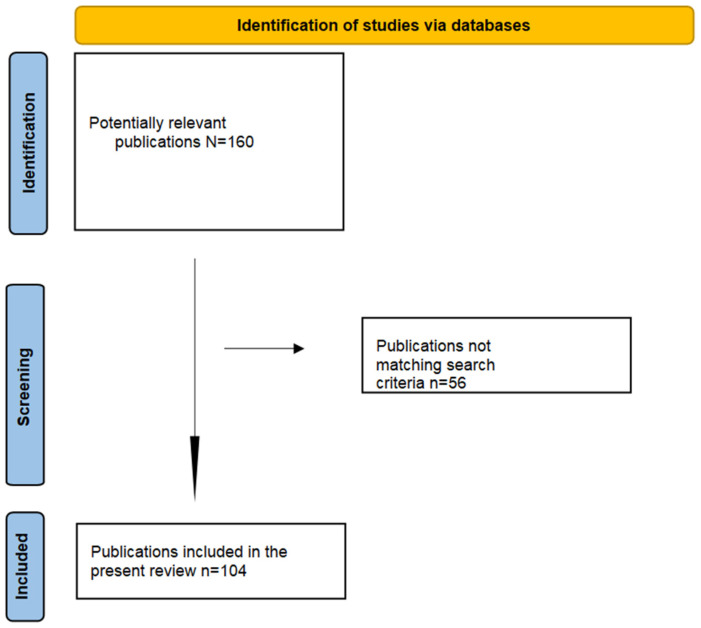
A flow diagram of the selection process is shown.

This study was conducted using the major scientific platforms, such as Web of Science, PubMed, and Scopus in the period between August and October 2024. For the bibliographic search, the keywords used were the following: “breastfeeding”, “human milk oligosaccharides”, and/or “microbiota–gut–brain axis”, and/or “infants”, and/or “malnutrition”. In our review, we include original papers and include the most recent papers. The inclusion criteria in our study were (i) original research papers that focus on HM, (ii) human and/or animal studies that focus on the health benefits in the microbial environment in HM, and the gap with IF for neurodevelopment in newborn (iii) meta-analyses. The exclusion criteria were the following: (i) case reports, (ii) commentaries, and (iii) articles that were not in English. Through the established search strategy, 160 articles were found, of which 104 were relevant and included in this article. A flow diagram of the selection process is shown in Figure 1.

## 3. Results

### 3.1. Human Milk Composition

HM is currently considered the gold standard of neonatal nutrition, as it is rich in bioactive components; prebiotics (HMOs); immune factors (e.g., immunoglobulins, lysozyme, and lactoferrin); and macro and micronutrients that accompany the growth of the newborn [11]. The intake of breast milk is recommended by the World Health Organization (WHO) up to the sixth month of life, then weaning should begin with 50% milk and new foods. However, while breastfeeding is also called “personalized medicine” because it is effective in preventing deaths from infant mortality, the percentage of breastfed children in low- and middle-income countries remains very low [12]. For this reason, the WHO and the Global Nutrition Target (GNT) are committed to implementing global nutrition policies to reduce the prevalence of infant mortality due to poor breastfeeding.

HM, a personalized medicine for each newborn, is essential for its growth, as it is rich in nutrients and bioactive substances. HM changes composition to meet the energy demands of the newborn during its growth. After birth, between the first two to four days, colostrum is produced; colostrum is rich in proteins and minerals such as Fe, Cu, and Zn (iron copper and zinc) but has low levels of fat and lactose [13]. Then, from the fifth day to two weeks postpartum, transitional milk will be secreted, very similar in composition to colostrum. From two weeks onwards, what will be secreted by the mammary gland is mature milk. Mature milk is composed of carbohydrates (7%), of which the most abundant is lactose, fats (3.8%), and proteins (1%), of which the most abundant are casein, lysozyme, lactoferrin, and immunoglobulins (IgAs) [14]. The main source of carbohydrates is lactose, a disaccharide composed of glucose and galactose, which promotes brain development [15]. In addition, fatty acids present in HM, such as long-chain polyunsaturated fatty acids (LCPUFAs), are strongly influenced by maternal nutrition, and are involved in neuroimmunomodulatory mechanisms in newborns [16]. Moreover, growth factors present in HM such as nerve growth factors (NGF) and brain-derived neurotrophic factor (BDNF) can promote neural maturation and growth [17].

**Figure 2:**
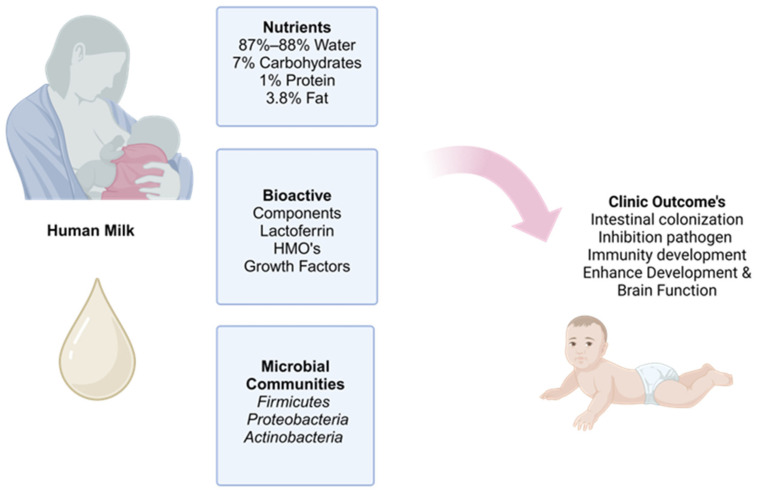
Composition of breast milk and clinical outcomes in newborns.

In addition to macronutrients, HM is rich in bioactive compounds with immuno- and neuromodulatory properties (Figure 2). Colostrum appears to have more bioactive components than mature milk; they have immunoglobulins, growth factors, antimicrobial peptides, and HMOs. HMOs prevent the adhesion of pathogenic bacteria to the mucosal layer. Recent studies show that *Bifidobacterium longum* ssp. infantis is highly expressed in breastfed newborns, thus improving their intestinal eubiosis. Furthermore, HMOs seem to have an antimicrobial role against Group B Streptococcus (GBS). The latter causes chorioamnionitis, infection, and inflammation of the amniotic fluid, placenta, and fetus, with a consequent risk of preterm rupture of membranes and preterm labor [18]. Furthermore, once HMOs have reached the intestinal lumen and entered the systemic circulation, they can reach the blood–brain barrier, promoting synaptogenesis and neurodevelopment of the infant [19]. Moreover, recent studies have shown that N-acetylneuraminic acid (Neu5Ac) and 2′-fucosyllactose (2′-FL) are significantly associated with synaptic development in infants at 1 month of age and with better motor and cognitive scores at 6 months [20].

However, the concentration of bioactive components in HM can vary as a result of maternal nutrition, lifestyle, and environmental factors [21,22,23]. In fact, RW Dabeka et al. demonstrated that cadmium levels (a toxic agent present in tobacco and a potential food contaminant) in HM were strongly correlated with maternal exposure to cigarette smoke (*p* = 0.005) [24,25]. Furthermore, the presence of persistent organic pollutants (POPs), heavy metals, and pesticides also seems to have the ability to accumulate in breast milk [26]. However, although environmental exposure can influence the composition of breast milk, the studies conducted to date have shown that the composition of the milk itself and its immunological factors can mediate the potential damage from environmental pollution [27]. However, to date, HM is the best source of nutrition for the growth of newborns and their neurodevelopment.

### 3.2. Preterm Milk Composition

Preterm infants, compared to healthy full-term infants, are born with a risk of short- and long-term complications, nutritionally compromised, and with a still immature gastrointestinal tract [28]. The recommended diet is through breast milk, which, however, due to the macronutrient content, will not be sufficient to meet the growth requirements of the preterm infant. For this reason, it is necessary to resort to milk fortification. The European recommendations on nutritional intake levels aim at 110–135 kcal/kg per day, with 4.0–4.5 g of protein/kg per day for premature infants weighing <1000 g and 3.5–4.0 g of protein/kg per day for infants weighing between 1000 and 1800 g [29]. Moreover, adequate amounts of calcium and vitamin D are essential for the growth of preterm infants. Most of these infants in the NICU (Neonatal Intensive Care Unit) require enteral and parenteral feeding. However, multi-nutrient fortification based on calcium, phosphorus, and vitamin D of breast milk is the ideal source of nutrition to ensure proper growth and development [30].

Preterm milk is richer in bioactive components such as immunoglobulin, epidermal growth factor, HMOs, fat, protein, amino acids, copper, and zinc; however, it is more deficient in calcium [31]. Recent studies have shown that, in the colostrum of mothers who gave birth prematurely, there were lower levels of docosahexaenoic acid (DHA) and higher levels of arachidonic acid (AA) [32]. These unsaturated fats are essential, because they participate in the synthesis of cell membranes, particularly in the brain and retina. Also, Ann Hellström et al. demonstrated that low levels of DHA and AA in preterm infants are associated with higher levels of Interleukin (IL)-6 in cord blood (*p* < 0.001). IL-6 is a pro-inflammatory cytokine, which is also found to be very high in very low birth weight (VLBW) infants [33]. Moreover, several studies suggest that preterm milk is naturally richer in protein and can reach levels as high as 4.1–2.1 g/100 mL, as opposed to protein in term milk, which is 1.2 g/100 mL [34].

Furthermore, in premature infants, the risk of developing NEC, which typically occurs between 2 and 6 weeks of life, can lead not only to a higher risk of mortality and morbidity but also to serious neurological developmental outcomes, as the pro-inflammatory state characterizing NEC favors the release of free radicals that, by damaging pre-oligodendrocytes, cause brain damage. Even in these cases, breastfeeding has been shown to reduce the probability of developing NEC by 6–10 times compared to formula-fed premature infants. In fact, IgA, HMOs, and the breast milk microbiome appear to play a role in protecting the mucosal barrier, protecting against pro-inflammatory and dysbiosis states and thus preventing the risk of developing NEC. In fact, in a prospective double-blinded RCT conducted by Serce Pehlevan O et al., they demonstrated that, on a sample of infants with VLBW (<1500 g), they received formula feeding supplemented with a mixture based on *L. rhamnosus*, *L. plantarum*, *L. casei*, and *B. lactis* plus HMOs, reducing the incidence of NEC in the sample (summarized in Table 1).

However, to date, studies are still underway to find the best growth monitoring methods and nutritional assessment for preterm and VLBW infants.

### 3.3. Human Milk Microbiome and the Gut–Mammary Gland Axis

The discovery of microorganisms in breast milk is not a recent discovery. Still, if initially, the organisms present in it were attributed only to a potential danger due to the transmission of maternal–infant infections through breastfeeding, today, we know that the microbes present there represent much more. The presence of a peculiar microbiota in breast milk and the richness of commensal and symbiotic bacteria represent a source of probiotics, which has led to a growing interest in the scientific panorama for its potential benefits and the methods of transmission during breastfeeding [49]. Microorganisms present in HM appear to benefit not only the newborn but also the mother, preventing breastfeeding mastitis, and in newborns, they promote intestinal colonization.

Beyond the interindividual variations found in the composition of milk, including its microorganisms, recent studies have shown that there is a common core in the presence of specific genera such as *Staphylococcus*, *Streptococcus*, and *Propionibacterium* [50]. However, thanks to advanced next-generation sequencing (NGS) studies, anaerobic bacteria such as *Bacteroides, Blautia, Clostridium, Roseburia*, and *Ruminococcus* have been identified [51]. The latter are species that live in the intestine; for this reason, this discovery has favored the thought that the microbial species present in human milk can populate a newborn’s intestines, thus starting their colonization. Some studies explain this finding thanks to the connection between the intestine and the mammary gland. Arroyo et al. showed how the intake of oral probiotics in breastfeeding women transfers the same phyla into the milk itself [52]. These findings suggest that the presence of an entero-mammary pathway allows bacteria present in the maternal gut to travel to the mammary gland and be taken up by the infant through the first breastfeeding.

However, the presence of microorganisms in breast milk, as well as in our intestine, seems to depend strongly on the mother’s health, her diet, and the use of antibiotics during breastfeeding. Solo et al. demonstrated that, in a cohort of healthy women (*n* = 160) subjected to antibiotic therapy, there was a reduction in *Bifidobacteria* (important because they are able to ferment HMOs). It is also known that the maternal body mass index (BMI) before and during pregnancy can influence not only the health status of the newborn but also the microbial composition of the milk. Reduced microbial biodiversity has been observed in women with a high BMI (overweight/obesity), starting from the composition of the colostrum [53]. However, further studies are needed to better understand the mechanisms of transmission between the gut–mammary gland and human milk.

### 3.4. Human Milk: A Major Drive in Brain Development and Growth

If more than fifty years ago, it was common opinion that people’s health was determined exclusively by their genetic heritage obtained at birth and not influenced by environmental factors, due to the developmental origins of the health and disease hypothesis (DOHaD), this vision has changed [54], proposing how environmental exposure both in the prenatal and postnatal periods can influence both acutely and permanently the health of a newborn by “programming” the phenotype but not the genotype. From here is the importance of the first 1000 days of life, renamed “window of opportunity” [55].

It is in this window of opportunity that breast milk plays a crucial role. Numerous studies have shown how HM is associated with improved neurodevelopmental outcomes, even in preterm infants. Hosseini et al. showed how stem cells present in HM can differentiate into neurons, astrocytes, and oligodendrocytes [56]. Moreover, myelin, the sheath that surrounds neuronal axons and transmits an adequate speed of transmission of the action potential, also begins to form at the 32nd week of gestation and continues during the postnatal phase, in the first thousand days of life [57]. Sean C.L. Deoni et al. through a cross-sectional study, demonstrated, using magnetic resonance imaging (MRI) in (*n* = 133) healthy children aged 10 months to 4 years exclusively breastfed for at least 3 months how the microstructure of the white matter is positively correlated with the duration of breastfeeding and with an improvement in behavioral and cognitive measures [58]. Furthermore, in the postnatal phase, a newborn’s microbiota can produce molecules that reach the brain through the circulatory system and support its neurodevelopment, determining a bidirectional communication thanks to the MGBA. The connection between the gut and the brain is also heavily investigated for pathologies such as autism spectrum disorder (ASD), epilepsy, and anxiety disorder [59,60,61]; it has been reported that children with ASD have gastrointestinal disorders with often severe dysbiosis, such as leaky gut syndrome [62,63]. Moreover, recent studies have shown that no breastfeeding or mixed breastfeeding in favor of IF is associated with an increased likelihood of ASD diagnosis [64]. However, what they have in common is the developmental trajectories of a newborn, their neurodevelopment, and the settlement of the bacteria that will accompany them in the years to come being adequate nutrition. In fact, iron, zinc, and iodine deficiencies can compromise the neurodevelopment of the newborn, as well as inadequate breastfeeding and weaning [65].

## 4. Discussion

### 4.1. Human Milk, Infant Formula, and Donor Milk: What We Know

Health inequalities are defined as the disparity in health status between people and populations as an inevitable consequence of genetic and socioeconomic differences or individual choices following cultural, religious, and social elements [66].

Globally, social and behavioral change interventions (SBCs) are constantly implemented and strengthened to reduce maternal and child mortality and morbidity [67]. This is also done through breastfeeding education as a primary source of health for a newborn and beneficial effects on the mother. However, breastfeeding can often fail, following cultural factors such as education, husband’s disapproval, and other psychosocial influences [68]. In countries such as Italy, Germany, and the United Kingdom (according to a UNICEF report), the breastfeeding rate ranges from 81% to 86%. Much higher are the percentages of Northern European countries that reach 92–98%. In poor countries, wealthy women are less likely to breastfeed, while, in rich countries, poorer women breastfeed less [69,70]. Improving exclusive breastfeeding and establishing intervention policies should therefore be a national and international priority.

However, if breastfeeding is not possible, we can refer to donor milk or IFs. The World Health Organization and the American Academy of Pediatrics (AAP) have recommended the use of pasteurized donated human milk since 2016, if the mother’s milk is not available for newborns under <1500 g and for preterm newborns [71]. However, this milk does not contain an adequate amount of proteins for a preterm; for this reason, it must be fortified with multi-nutrient formulas available in powder or liquid form [72,73]. Moreover, donated milk must undergo a pasteurization process (subjected to temperatures of 62.5 °C × 30 min). It is subject to a depletion of enzymes, bioactive compounds, immunoglobulins, and microorganisms, compromising the optimal benefits of the milk itself. Tyson et al. demonstrated that infants with VLBW (<1500 g) and fed with unfortified donor milk grew more slowly in head circumference, weight, and length than preterms fed with enriched formula. In another study entitled DoMINO (Donor Milk for Improved Neurodevelopmental Outcomes), they randomized VLBW infants to receive donor fortified milk (*n* = 181) and a group who received preterm formula (*n* = 182) for 90 days. No differences were observed in terms of the anthropometric measures [74]. However, for the most vulnerable infants, such as those born preterm or VLBW at birth, the WHO recommends the safe use of donor human milk. However, due to sociodemographic factors or poor national awareness and sensitization policies, not all vulnerable infants may have access. The aim to address these challenges is therefore to establish global policies that can ensure equitable access and to understand the potential barriers for donors better [75].

In addition to donor milk, IF are also designed as substitutes for breast milk and formulated to reduce the differential gaps between breast milk and IF.

IF is available in three formulations, namely powdered, liquid, and ready-to-use (the most expensive formula, as it does not require mixing by the consumer). The composition of these three preparations is mainly derived from bovine milk, which has a higher fat (4.4–6 g/100 kcal) and protein (1.8–2.5 g/100 kcal) content than HM; this excess of protein in infants seems to correlate with a higher probability of developing obesity in later years. Furthermore, to cover the need for pre- and probiotics in IF, these are supplemented to enrich the intestinal microbiota of the infant. The study by Arslanoglu et al. demonstrated that infants fed with IFs enriched with FOS (fructo-oligosaccharides) and GOS (galacto-oligosaccharides) had a lower rate of recurrent infections, with a consequent reduction in antibiotic protocols [76]. Furthermore, in infants at risk of atopy, the supplementation of IF enriched with GOS (*n* = 201) prevented the development of asthma and respiratory tract infections in the first two years of life [77]. For this reason, further studies investigating the capacity of probiotics in their immunomodulatory effect in atopic disease and food allergy are needed [78]. Furthermore, the administration of IF enriched with *B. lactis* Bb-12 in newborns (0–6 months) born by C-section showed levels of fecal *Bifidobacteria* similar to those born by C-section but breastfed [79].

However, if, in term newborns, the efficacy of probiotic taxa is widely studied, strong evidence is still lacking for premature newborns, for whom the degree of acute inflammation and risks of NEC, sepsis, and dysbiosis can make the clinical picture more complicated. In fact, the Food and Drug Administration (FDA) warns that bacteria in probiotics have been reported in scientific evidence to cause bacteremia, with risks of serious development in very premature and very low birth weight (VLBW) infants [80].

For these reasons, which fortified formula or which supplementation in prebiotics and probiotics is more suitable for preterm infants remains a much debated question, on which future scientific research is needed.

### 4.2. Postnatal Microbiome and Nutrition Affect Adult Health

Malnutrition is an endemic phenomenon that affects the entire world population; malnutrition by defect, micronutrient deficiencies, overweight, and obesity are a global health risk with strong disparities between countries. There are 149 million children (aged < 5 years) whose growth is slowed, while 40.1 million are overweight [81]. For this reason, nutritional healthcare interventions are essential in the pediatric population and early stages of pregnant women.

Healthy eating and correct health interventions in the preconception period are necessary to ensure the right intake of micro and macronutrients and the health status for both a pregnant woman and her fetus [82,83,84]. For example, it has been shown that delayed clamping of the umbilical cord improves iron levels and neurological development in full-term newborns [85]. In preterm newborns, however, Kangaroo Mother Care (KMC) with early breastfeeding has shown a greater weight gain by the 14th day of life [81]. Moreover, KMC and mother–infant skin-to-skin contact seem to have a role in positively altering the neonatal microbiota [86,87]. A pilot study conducted in NICU on preterm infants (*n* = 30) at <32 weeks of gestation showed that KMC led to a reduction of Escherichia (*p* = 0.05) [87].

However, longitudinal and cohort studies are needed to shed more light on this aspect.

What is increasingly evident, however, is the increasing prevalence of malnutrition in school age children and adolescents [88]. Epidemiological estimates indicate a 30% prevalence in the BMI above the 85th percentile in the United States, and the European pediatric population with obesity and overweight is also increasing [89]. However, nutrition in childhood will determine the adult phenotype and its microbiota.

Childhood overweight and obese are related to the development of dyslipidemia, hypertension, type 2 diabetes mellitus (DM2), and non-alcoholic fatty liver disease (NAFLD) in adulthood [90,91,92]. Scientific studies to date show a strong connection between microbial composition and host phenotype. One of the first studies conducted by Jeffrey Gordon et al. and published in science showed how mice that received the fecal microbiota of an obese twin showed a greater fat mass than mice that received the intestinal microbes of a lean twin [93]. Furthermore, an altered microbiota will have potential outcomes on mood, cognition, and anxiety [94,95,96]. Moreover, a study by Fiorentino et al. demonstrated how, in patients with autism spectrum disorder (ASD), the integrity of the blood–brain barrier (BBB) is closely linked to that of the mucosal barrier at the intestinal level; in fact, in ASD patients, there are elevated levels of claudin (a family of proteins that represent the paracellular barrier at the epithelial level) at the brain level compared to healthy controls [97].

What is certain today is the ability of this “dynamic organ” to intervene, regulate, and modulate numerous pathways of our organism, contributing to the state of health and disease. However, more in-depth, long-term future studies are needed, with a population stratification based on age and clinical picture.

## 5. Conclusions

In conclusion, the MGBA is a dynamic system capable of influencing the health of the host starting from the fetal microenvironment. One of the greatest drivers of this modulation is the nutritional regime. It is therefore important to promote health interventions that guide the population and the medical health personnel themselves in preventing the risk of diseases, starting from maternal–infant health through the promotion of correct nutrition with functional foods to promote the correct cross-feeding of the microbial niches of pregnant women, promote breastfeeding when possible, and the use, if necessary, of specific probiotic strains that have strong scientific evidence.

## Figures and Tables

**Table 1:** Summarizing the different bioactive components in HM and their effects on neurodevelopment and gut health in preterm and term infants.

Author/Year	Population	Type of HMO’s	Out Comes
Montagne et al. 1998 [28]	Preterm/term	Higher HMO in preterm	N.A.
Embleton et al. 2023 [29]	Preterm	DSLNT	Protective for NEC
Cheema et al. 2022 [30]	Term	DFLNH and LNnT	Protective for fat (obesity)
Cheema et al. 2022 [30]	Term	2′-FL, 3′-FL, DFLac, DFLNH and LSTb	Increase body composition
Binia et al. 2021 [31]	Term	3′-SL	Weight for length increases
Lagström et al. 2020 [32]	Preterm/term	LNnT	Negative related to height and weigh
Jasmine et al. 2017 [33]	Term	3′-SL	Positive impact on weigh for age
Matsuki et al. 2016 [34]	Term	2′-FL	Increase *Bifidobacteria* spp.
Lewis et al. 2015 [35]	Term	2′-FL	Increase *Bifidomacteria* spp.
Dogra et al. 2021 [36]	Term	2′-FL and LNnT	Decrease risk of Respiratory infections
Autran et al. 2018 [37]	Preterm	DSLNT	Negative related with NEC
Morrow et al. 2011 [38]	Preterm	FUT2	Low secretor associated with NEC; non secretor associated with gram negative sepsis
Masi et al. 2021 [39]	Preterm	DSLNT	Negative related with NEC
Wejryd E 2018 [40]	Preterm	LNDFH-I LSTa	Negative related with NEC Positive related with growth
Stepans et al. 2006 [41]	Term	LNFP-II	Protective to respiratory and gastrointestinal infections
Torres et al. 2020 [42]	Preterm	FDSLNH	Protective to late onset neonatal sepsis
Morrow et al. 2014 [43]	Term	2′-FL LNDFH-I	Decrease risk of Campylobacter diarrhea Decrease risk of calicivirus diarrhea
Sprengel et al. 2017 [44]	Term	2′-FL	Protective to IgE mediated allergy
Cho et al. 2021	Term	3′-SL	Improve cognition and language function
Berger et al. 2020 [45]	Term	2′-FL	Improve cognitive development
Oliveros et al. 2021 [46]	Term	2′-FL; 6′-SL	Improve motor and cognitive development
Jorgensen et al. 2020 [46]	Term	holistic fucosylated and sialylated HMOs	Improve linguistics performances
Rozé et al. 2022 [47]	Preterm	LNFPIII	Improve neurodevelopmental
Ferreira et al. 2021 [48]	Term	LNT	Improve neurodevelopmental

HMOs: human milk oligosaccharides; DSLNT: disialyllacto-N-tetraose; DFLNH: difucosyllacto-N-hexaose; LNnT: lacto-N-neotetraose; 2’-FL: 2’-fucosyllactose; 3’-FL: 3’-fucosyllactose; DFLac: difucosyllactose; LSTb: sialyl-lacto-N-tetraose b; 3‘-SL: 3′-sialyllactose; LNFP I: lacto-N-fucopentaose I; FUT2: galactoside alpha-(1,2)-fucosyltransferase 2; LNDFH-I: lacto-N-difucohexaose; LSTa: sialyl-lacto-N-tetraose a; LNFP-II: lacto-N-fucopentaose II; FDSLNH: fucodisialyllacto-N-hexaose; 6’-FL: 6′- fucosyllactose; LNFPIII: lacto-N-fucopentaose III; LNT: lacto-N-tetraose.

## Data Availability

Not applicable.
